# Surgical Effectiveness of Uniportal-VATS Lobectomy Compared to Open Surgery in Early-Stage Lung Cancer

**DOI:** 10.3389/fsurg.2022.840070

**Published:** 2022-03-04

**Authors:** Dania Nachira, Maria Teresa Congedo, Diomira Tabacco, Carolina Sassorossi, Giuseppe Calabrese, Mahmoud Ismail, Maria Letizia Vita, Leonardo Petracca-Ciavarella, Stefano Margaritora, Elisa Meacci

**Affiliations:** ^1^Department of General Thoracic Surgery, Fondazione Policlinico Universitario “A. Gemelli,” Istituto di Ricovero e Cura a Carattere Scientifico (IRCCS), Università Cattolica del Sacro Cuore, Rome, Italy; ^2^Department of Thoracic Surgery, Klinikum Ernst von Bergmann Potsdam, Academic Hospital of the Charité-Universitätsmedizin Humboldt University Berlin, Potsdam, Germany

**Keywords:** NSCLC, Uniportal-VATS, nodal upstaging, disease-free survival (DFS), oncological outcomes

## Abstract

**Background:**

Although the feasibility and safety of Uniportal-Video-Assisted thoracic surgery (U-VATS) has been proven, its surgical effectiveness is still debated. The aim of this study is to assess the equivalence of the U-VATS approach compared with an open technique in terms of surgical (nodal-upstaging, complications, and post-operative results) and short-term survival outcomes.

**Methods:**

The clinical data of patients undergoing lobectomy for NSCLC at our center, from January 2014 to December 2019, were analyzed retrospectively. All patients undergoing open or U-VATS lobectomy with lymphadenectomy for early-stage lung cancer (cT1-T3N0, stages IA-IIB) were included in the study. Only 230 patients satisfied the inclusion criteria. Group bias was reduced through 1:1 propensity score matching, which resulted in 46 patients in each group (open surgery and U-VATS).

**Results:**

The intra- and post-operative mortality were null in both groups. There was no difference in the post-operative complications (*p*: 1.00) between U-VATS and open lobectomy. There was also no recorded difference in the pathological nodal up-staging [11 (23.9%) after thoracotomy vs. 8 (17.4%) after U-VATS, *p*: 0.440). The chest tube duration was longer in the open group (*p*: 0.025), with a higher post-operative pain (*p:* 0.001). Additionally, the 3-year overall survival (OS) was 78% after U-VATS lobectomy vs. 74% after open lobectomy (*p*: 0.204), while 3-year disease-specific survival (DSS) was 97 vs. 89% (*p*: 0.371), respectively. The 3-year disease-free survival (DFS) was 62% in the U-VATS group and 66% in the thoracotomy group, respectively (*p*: 0.917).

**Conclusions:**

Uniportal-VATS lobectomy for the treatment of early-stage lung cancer seems to be a safe and effective technique with similar surgical and short-term survival outcomes as open surgery, but with lower post-operative pain and shorter in-hospital stay.

## Introduction

An increasing number of articles have been showing the role and effectiveness of Uniportal—Video-Assisted thoracic surgery (U-VATS) in performing more and more complex and technically demanding procedures, compared to traditional techniques, such as thoracotomy or triportal-VATS. Nevertheless, these studies are mainly retrospective, not randomized, single-institution researches (1–6).

Works evaluating the surgical outcomes of U-VATS are still few, also due to the quite recent introduction of the technique in the surgical field.

In thoracic surgery, besides the overall and disease-free survivals, an important surgical quality marker is the nodal upstaging (7), which in early-stage non-small cell lung cancer (NSCLC) (<IIB stage) can occur in about 20% of patients (8). Nodal upstaging is defined as the pathological finding of nodal metastases, hilar (pN1), or mediastinal (pN2), not expected according to the pre-operative evaluation and clinical staging (7). Therefore, the incidence of nodal upstaging is considered a criterion of quality and radicality of lymphadenectomy (LND) that has important implications on prognosis and adjuvant treatments.

Video-Assisted Thoracic Surgery (VATS) lobectomy is currently recommended over thoracotomy for the treatment of IA- IIB NSCLC because it was proven to guarantee similar long-term survival with less post-operative pain and shorter hospital stay (9).

However, if the efficacy of VATS LND is still debated, the adequacy of the U-VATS approach in performing LND compared to standard techniques has not been proven yet. The aim of this article is to assess the equivalence of U-VATS lobectomy in terms of surgical outcomes (like nodal-upstaging, complications, and post-operative results) and short-term survival outcomes compared to traditional open surgery.

## Materials and Methods

**Figure 1:**
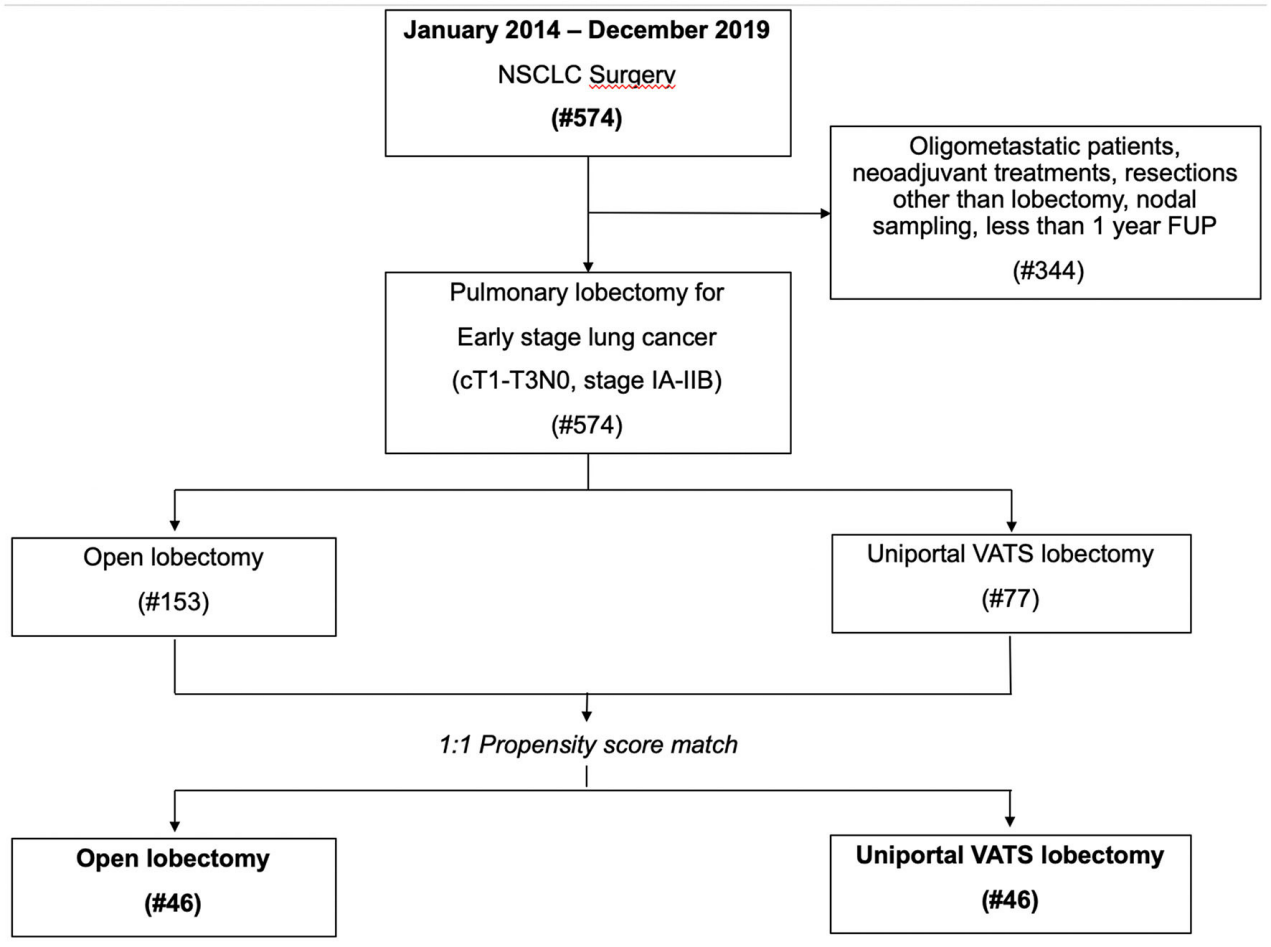
Study flow-chart with reasons for patient exclusion.

The clinical data of 574 patients undergoing lung resection for NSCLC at our Department from January 2014 to December 2019 were retrospectively reviewed. Among these patients, only 230 patients undergoing an open or U-VATS lobectomy with a radical LND for early-stage lung cancer (cT1-T3N0, stages IA-IIB) satisfied the inclusion criteria for the study. Patients operated on for diseases other than NSCLC, oligometastatic patients, or undergone neoadjuvant treatments, extended surgery, resections other than lobectomy or nodal sampling, or having <1-year follow-up were excluded to eliminate any possible confounding factor. The clinical records of all 230 patients were reviewed (Figure 1), in particular all details regarding the clinical and pathological stage of cancer, histology, centrality, the diameter of the lesion, the number of lymph nodes retrieved, type of approach, and lung resections, time of surgery, intra- and peri-operative complications, chest-tube duration, hospital stay, post-operative pain, and follow-up information (like mortality, recurrence and any adjuvant treatment, etc.) were analyzed.

Group biases were reduced through a 1:1 propensity score matching.

All patients signed informed consent for the treatment of their clinical data before surgical operation. This study was approved by the Institutional Review Board (I.R.B.) (approval n°43754/20 Prot. ID.3553) and was therefore performed in accordance with the ethical standards of the Declaration of Helsinki and its later amendments.

### Pre-operative Evaluation and Staging System

The pre-operative evaluation of the patients who were candidates for surgery included: routine blood tests, electrocardiography, radiological, and diagnostic examinations [total body CT, PET—CT, endobronchial ultrasound (EBUS) with nodal biopsies…], and pulmonary function test.

Invasive staging of the mediastinum with EBUS biopsies was carried out in each case where CT-scan and/or PET-CT showed evidence of nodal involvement in order to clarify the real extension of the disease or if the tumor was >3 cm or located close to the hilar structure, according to the 2014 European Society of Thoracic Surgeons (ESTS) guidelines for pre-operative mediastinal lymph-node staging (10). The 8th Tumor, Node, Metastasis (TNM) edition for lung cancer classification was adopted for clinical and pathological staging.

### Surgical Techniques

All procedures were performed under general anesthesia and single-lung ventilation, with the patient lying on the lateral decubitus. For all surgeries, the first operator was one of the two surgeons with the widest experience in open (at least 15 years) and U-VATS surgery in our team. Patients who had undergone surgery from January 2014 to May 2016 were operated on by thoracotomy. The U-VATS program started in our center in June 2016 and, since then, all patients affected by stage IA-IIB lung cancer were operated on by the U-VATS approach, progressively. Therefore, the open approach, still used for more central and big lesions in the first months of our U-VATS program, was subsequently abandoned for the treatment of early-stage lung cancer.

**Figure 2:**
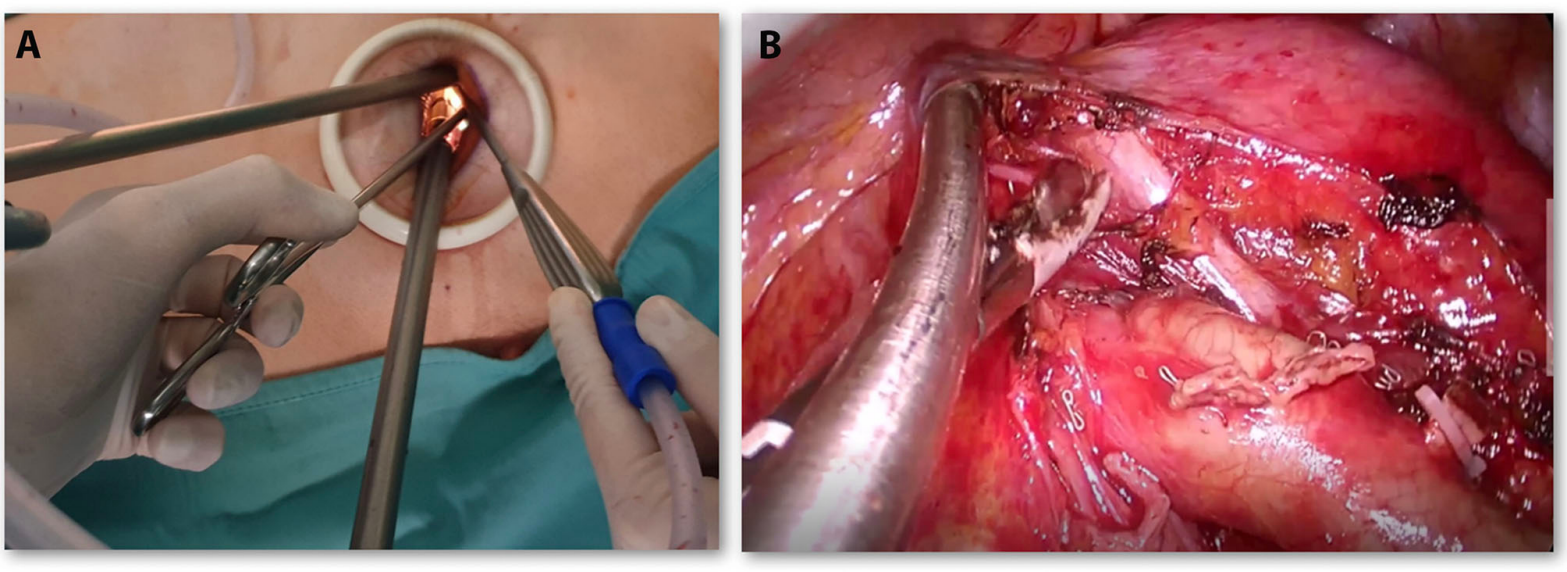
**(A)** Placement of endoscopic instruments through Uniportal—Video-Assisted thoracic surgery (VATS) incision. **(B)** U-VATS surgical view during the left side (station 5, 6) lymphadenectomy.

Open surgery was performed through a lateral muscle-sparing thoracotomy on the V intercostal space and usually, two chest tubes are left in place after surgery. U-VATS was carried out through a 4-cm single incision on the V intercostal space, with only one chest tube in the same incision, according to our standardized procedure (11, 12) (Figure 2A).

Lymphadenectomy (LND) was performed according to the oncological standard of radical LND in both techniques: lobe-related nodal stations 10 and 11 and nodes in positions 7, 8, and 9, as well as in 2, 3, and 4 for the right side, and 5 and 6 for the left side (Figure 2B) were removed.

### Statistical Analysis

The normal distribution of data was checked using the Kolmogorov–Smirnov test. Categorical variables were reported as numbers (%). Continuous variables were expressed as mean ± SD, or medians if non-normally distributed. Categorical variables were compared by Chi-square test; continuous variables by the independent- sample Student's *t*-test or the Mann–Whitney *U*-test, as appropriate. Univariate linear regression was performed to evaluate any possible risk factor for nodal upstaging. Any variable with a *p* < 0.20 at univariate analysis was included in the multiple regression model to detect the independent risk factors for lymph nodal upstaging.

Given the retrospective nature of the study, the open and U-VATS groups were not randomized but time-dependent, as expressed in the methods. To overcome this selection bias, a 1:1 propensity score, using the nearest neighbor matching method, was first performed to balance the baseline characteristics of the two groups. The variables included in the propensity score model were those that might have influenced the clinical decision on the surgical approach at the beginning of our U-VATS experience: age, gender, smoking habits, Chronic obstructive pulmonary disease (COPD), cardiovascular diseases, forced expiratory volume in 1 s, forced vital capacity, tumor dimension, and centrality of the tumor.

Survival analysis was performed by the Kaplan–Meier plot and log-rank test. Overall survival (OS) and disease-specific survival (DSS) were defined as the time elapsed between surgery and death of any cause or cancer, respectively. Disease-free survival (DFS) is the time between surgery and first disease recurrence.

A *p* < 0.05 was considered statistically significant.

Statistical analysis was performed using the IBM SPSS Statistics for Macintosh, Version 25.00 (Armonk, NY, USA).

## Results

Among the 230 patients, 149 (64.8%) were males, 66 (28.7%) were active smokers (with a pack-year of 30.66 ± 30.47), and 33 (14.3%) were ex-smokers. The mean age of the population was 68.83 ± 7.94 years.

A total of 153 patients were operated on by thoracotomy and 77 by U-VATS.

The patients from both groups, operated on by open and U-VATS approaches, were comparable for the baseline clinical characteristics, except for more males (*p*: 0.009), active smokers (*p*: 0.012), and COPD patients (*p*: 0.029) and lower FEV1% (*p*:0.012), FVC% (*p*: 0.044), and larger tumors (*p*: 0.03) with a trend toward more central tumors (*p*: 0.066) recruited in the open group, as shown in Table 1.

**Table 1:** Clinical characteristics of the whole population.

**Variables**	**Thoracotomy (#153)**	**U-VATS (#77)**	***p*-values**
Male gender (%)	108 (70.6%)	41 (53.2%)	**0.009**
Age (years)	68.93 ± 7.84	68.62 ± 8.19	0.780
Active smoker (%)	45 (29.4%)	21 (27.3%)	**0.012**
COPD (%)	65 (42.5%)	21 (27.3%)	**0.029**
Diabetes (%)	29 (18.9%)	14 (18.2%)	0.922
Cardiovascular disease (%)	49 (32.0%)	16 (20.8%)	0.093
PCO_2_	40.76 ± 33.65	38.78 ± 7.34	0.628
PO_2_	87.74 ± 67.20	81.22 ± 15.54	0.423
FEV1%	87.00 ± 26.97	96.70 ± 22.12	**0.012**
FVC%	100.22 ± 29.48	109.01 ± 18.75	**0.044**
Tumor size (cm)	5.83 ± 3.64	2.38 ± 1.56	**0.030**
Central tumor/size > 3 cm	58 (37.9%)	22 (28.6%)	**0.066**
Histology (adenocarcinoma/squamous cell carcinoma)	115 (75.2%)/35 (22.9%)	65 (84.4%)/11 (14.3%)	0.275

*Bold values stands for: p-value ≤ 0.05*.

The main histology was adenocarcinoma, with 115 (75.2%) cases in the open group and 65 (84.4%) in the U-VATS group (*p*: 0.275).

After pathological staging, a nodal upstaging was recorded in 24 patients (15.68%) in the open group and 11 (14.3%) in the U-VATS group (*p*: 0.810). In particular, a pN1-nodal upstaging in 19 patients (12.4%) in the open group and 2 (2.6%) in the U-VATS group (*p*: 0.05), while a pN2-nodal upstaging in 6 (3.9%) vs. 9 (11.7%), respectively (*p*: 0.022).

At regression analysis, no independent risk factor for nodal upstaging was identified.

To reduce recruitment biases in the two surgical groups, a 1:1 propensity score-matched analysis was conducted, obtaining 46 patients eligible from each group. The main clinical characteristics of the patients belonging to the two matched groups are reported in Table 2.

**Table 2:** Clinical characteristics of the patients after propensity score matching.

**Variables**	**Thoracotomy (#46)**	**U-VATS (#46)**	***p*-values**
Male gender (%)	30 (65.2%)	29 (63.0%)	0.828
Age (years)	67.96 ± 8.09	68.96 ± 8.05	0.554
Active smoker (%)	12 (26.1%)	12 (26.1%)	1.000
COPD (%)	16 (34.8%)	17 (36.9%)	0.828
Diabetes (%)	9 (19.6%)	9 (19.6%)	1.000
Cardiovascular disease (%)	12 (26.1%)	14 (30.4%)	0.596
PCO_2_	45.89 ± 56.87	38.10 ± 8.98	0.372
PO_2_	100.23 ± 112.83	82.47 ± 12.57	0.302
FEV1%	88.44 ± 32.69	89.28 ± 18.74	0.880
FVC%	98.82 ± 37.88	104.07 ± 17.43	0.442
Tumor size (cm)	2.77 ± 1.45	2.64 ± 1.44	0.661
Central tumor/size > 3 cm	22 (47.8%)	16 (34.8%)	0.172
Histology (adenocarcinoma/squamous cell carcinoma)	36 (78.3%)/8 (17.4%)	37 (80.4%)/9 (19.6%)	0.355

After matching, the mean operative time for U-VATS lobectomy was 188.21 ± 53.86 min vs. 157.13 ± 42.00 min for the open one (*p*: 0.478). Intraoperative complications were null in both groups.

There was no difference in post-operative complications (nine cases in both groups, *p*: 1,00) between U-VATS or open lobectomy and the main complications recorded were grade I–II according to Clavien-Dindo classification (atelectasis, atrial fibrillation, and pneumonia) and 2-grade IIIB (one patient re-operated for bleeding in the U-VATS group and one in the open group).

Intra- and post-operative mortality was null in both groups.

The visual analog pain score (VAS) on the first post-operative day was lower in the U-VATS group compared to the Thoracotomy group (3.758 ± 1.22 vs. 7.55 ± 2.45, *p* << 0.001), similar to the number of patients who developed chronic paresthesia/neuralgia 15 days after surgery (2 vs. 15.43 ± 3.59, *p* << 0.001). Chest tube duration was longer in the open group (Mean: 5.93 ± 1.72 vs. 4.79 ± 2.09 days, *p*: 0.025; Median: 6 vs. 4 days, *p*: 0.003), resulting in a clinically longer post-operative hospital stay (Mean: 6.46 ± 1.93 vs. 5.70 ± 2.21 days, *p*: 0.08; Median: 6 vs. 5 days, *p*: 0.01).

At a pathological staging, the mean number of nodes retrieved in the thoracotomy and U-VATS groups were 15.50 ± 7.84 and 16.53 ± 8.66 (*p*: 0.785), respectively. In particular, the distribution of nodes removed per station was as follows: station 10: 2.33 ± 1.60 vs. 2.50 ± 1.37, *p*:0.13; station 11: 2.77 ± 1.85 vs. 3.00 ± 1.73, *p*: 0.34; station 3–4: 3.81 ± 1.22 vs. 4.25 ± 3.40, *p*: 0.67; station 7: 2.87 ± 2.03 vs. 3.00 ± 2.52, *p*: 0.83; station 8–9: 2.00 ± 1.41 vs. 3.00 ± 2.61, *p*: 0.52; station 5–6: 2.50 ± 1.22 vs. 2.00 ± 1.83, *p*: 0.82.

A nodal upstaging was found in 11 patients (23.9%) in the open group vs. 8 (17.4%) in U-VATS (*p*: 0.440); in particular, a pN1-upstaging was recorded in nine patients (19.6%) in the open group and 5 (10.9%) in the U-VATS group (*p*: 0.246), while a pN2-upstaging was recorded in two patients (4.3%) in the open group vs. 6 (13.0%) in U-VATS (*p*: 0.139). To reduce any confounding factors, the lymph node ratio (LNR), defined as the ratio between metastatic lymph nodes and all the dissected lymph nodes, was also evaluated. The N1-LNR in the open group was 0.33 ± 0.32 vs. 0.21 ± 0.20 in U-VATS (*p*:0.41), the N2-LNR was 0.12 ± 0.20 vs. 0.56 ± 0.42 (*p*: 0.03) and the total LNR was 0.22 ± 0.17 vs. 0.32 ± 0.21 (*p*: 0.25).

The univariate regression analysis on 92 patients after propensity score matching showed that the risk factors for nodal upstaging were the following: age over 70 years (*p*: 0.045) and adenocarcinoma histology (*p*: 0.014); the type of technique (open or U-VATS) was not a significant risk factor (*p*: 0.445). The multiple regression model confirmed that the only independent risk factor was age over 70 years (HR: 2.865, 95% CI [0.895–9.179], *p*: 0.041).

After surgery, three patients in the open group and two in U-VATS were lost at follow-up, which was in mean 36.02 ± 21.19 months and at least 12 months for all patients.

There was no difference between the two groups in the number of patients undergoing adjuvant therapy (*p*: 0.213) or having a recurrence of the disease (0.858).

**Figure 3:**
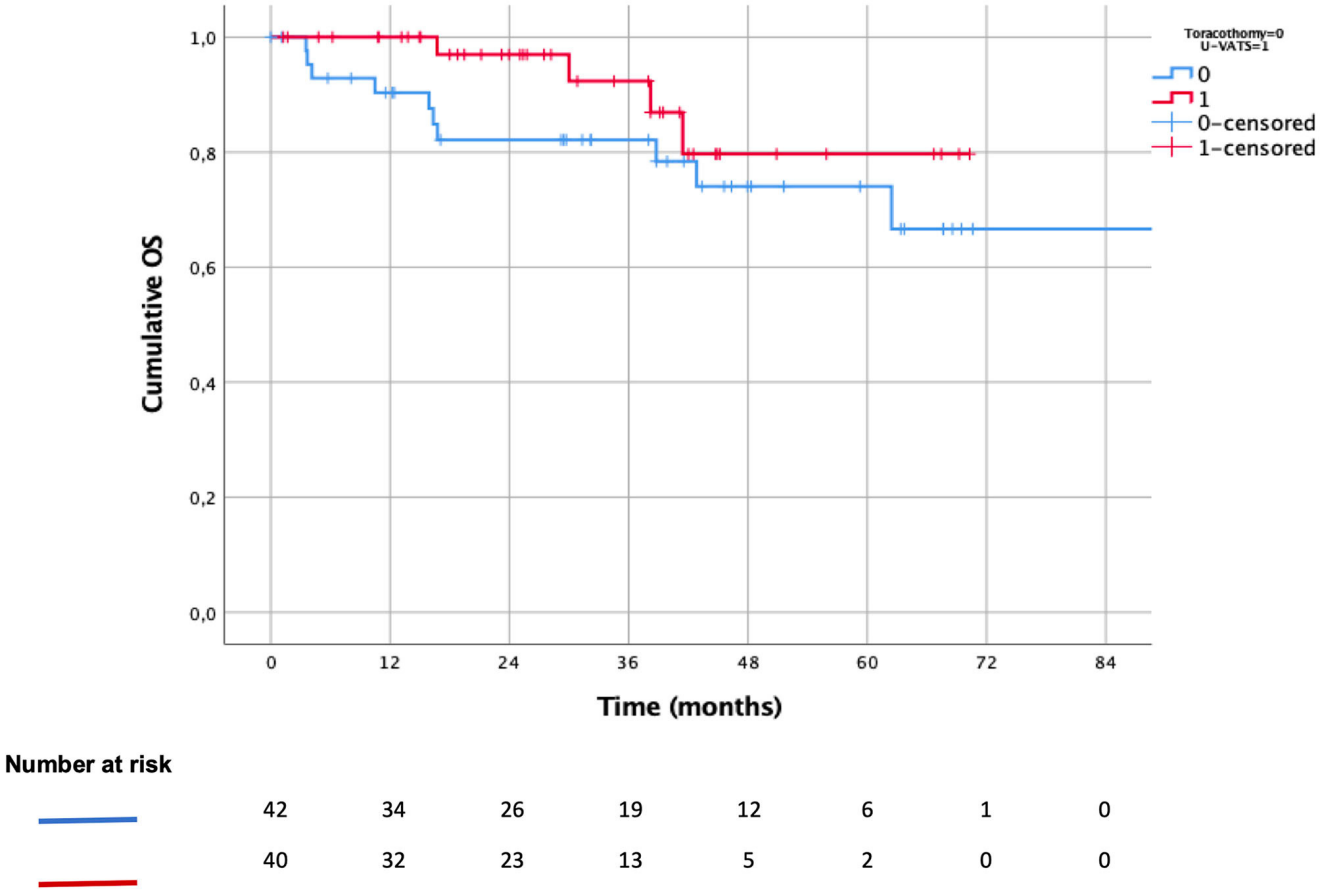
Kaplain–Meier overall survival (OS) analysis.

**Figure 4:**
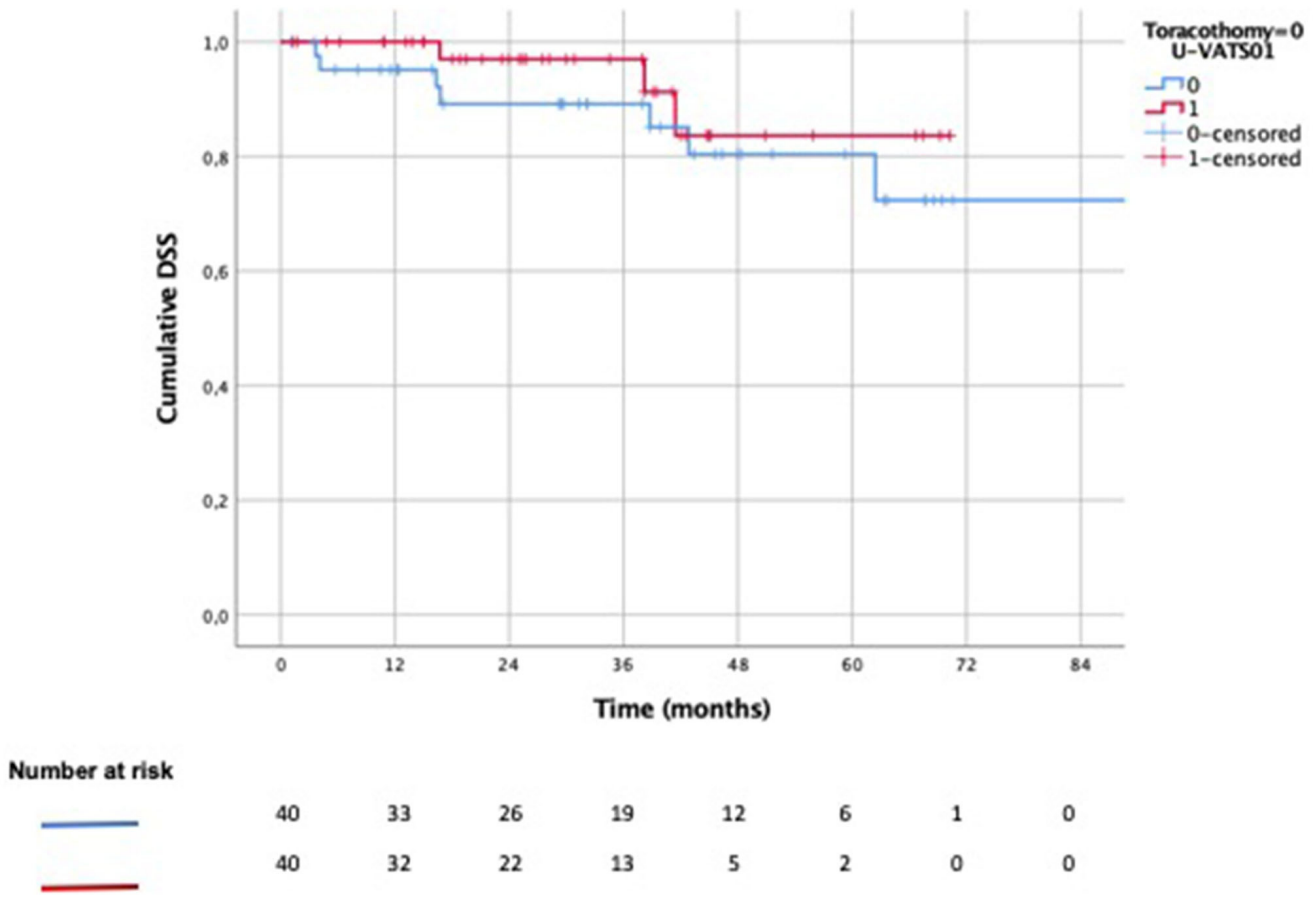
Kaplain–Meier disease-specific survival (DSS) analysis.

**Figure 5:**
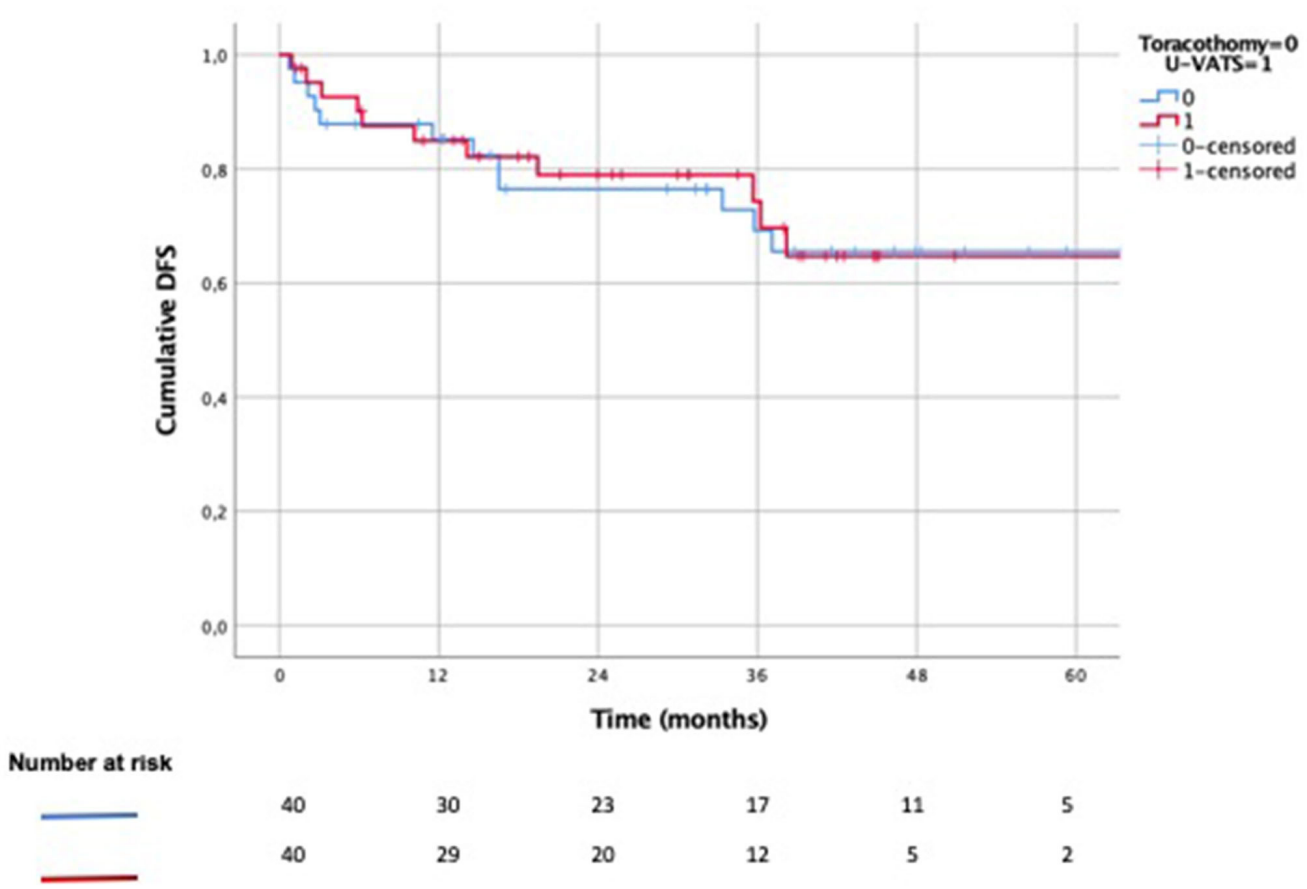
Kaplan–Meier disease-free survival (DFS) analysis.

The 3-year (OS) was 78% after U-VATS lobectomy vs. 74% after open lobectomy (Figure 3, *p*: 0.204), while the 3-year DSS was 97 vs. 89% (Figure 4, *p*: 0.371), respectively. The 3-year DFS was 62% in the U-VATS group and 66% in the thoracotomy group, respectively (Figure 5, *p*: 0.917).

## Discussion

First introduced in 1998 for diagnostic procedures (13), in the last years, U-VATS gained growing popularity and several centers in the world have been using it as the main approach for pulmonary lobectomies and complex procedures (14, 15).

Uniportal-Video-Assisted thoracic surgery (U-VATS) is a more ergonomic technique for surgeons, ensuring natural hand-eye coordination, improving body posture, and reducing neck overload (16).

Furthermore, it provides the same anterior approach as thoracotomy, with direct visualization of the target tissue, a good lung mobilization, and palpation.

Probably, for the reasons mentioned above, this approach seems to have a quite shorter learning curve for major lung resections compared to other minimally invasive techniques, above all, after attending dedicated courses and masterclasses (17, 18).

The single 3–4 cm incision (on 4th or 5th intercostal space), with no muscle disruption, no rib spreading, and no necessity of trocars (11), seems to be the major point of strength in reducing post-operative pain, allowing patients to have a faster recovery and shorter post-operative hospital stay, with good cosmetic results.

Uniportal-Video-Assisted thoracic surgery (U-VATS) is also less expensive than robotic surgery and it seems to have the same safety and surgical efficacy as other techniques (18). The main opponents of the technique are worried about the few solid clinical evidence on surgical and survival outcomes available till now.

In our center, we used the U-VATS approach for lung resections for NSCLC since 2016, and we have been having good results in terms of chest tube duration, post-operative pain, and hospital stay (15). Till now, few reports compared surgical outcomes after triportal-VATS and U-VATS (2, 4, 19): they found out that U-VATS was associated with significantly less intraoperative blood loss, less chest tube drainage volume and duration, and less post-operative pain and hospital stay. The same results were also reached by Wu and coworkers (20), recently. They also proved that, on a total of 443 patients who underwent VATS (197 U-VATS and 256 triportal-VATS), there were no significant differences in 1-,2-, and 3-year survival rates between the two groups of VATS and that the number of lymph-nodes removed per station was similar and consistent with required standards in the two groups (10).

### Minimally-Invasive LND

Lymphadenectomy (LND) has an important role in the correct pathological staging and predicting the prognosis of the patients (21). A recent article by Toker et al. (22) compared the effectiveness of LND after pulmonary lobectomy with VATS, robotic and open approach, concluding that all techniques had similar results in terms of lymph nodes retrieved; only robotic surgery seemed to guarantee a better removal of hilar nodes.

Nevertheless, there is an ongoing debate on the better minimally-invasive techniques in achieving an accurate LND in early-stage lung cancer.

Some meta-analyses (21) and reports concluded that the incidence of lymph-node up-staging was lower after VATS than after thoracotomy (23, 24). Martin et al. (25) reported a pN1-upstaging in 4.8% of cases in VATS compared to 9.9% in thoracotomy but with a higher long-term survival after VATS. Other authors (26) concluded that VATS is not inferior to other approaches in LND and gives better survival results.

In particular, some studies (27) evaluated the safety and effectiveness of U-VATS for systematic mediastinal lymph-node dissection compared to multi portal VATS. Liu et al. (27) analyzed U-VATS LND in 149 patients and compared it to multi portal VATS LND in 389 patients: they detected a lower number of lymph-nodes resected in the early stages of the U-VATS learning curve, while they showed no difference between the two approaches during the last phase of the learning curve. This aspect can be explained by the necessary training in U-VATS for achieving proper instrumentation for a good exposure of the mediastinal station (above all station 7 and station 6). The use of dedicated instruments, proper insertion of instruments through the incision, and use of energy devices can importantly facilitate U-VATS LND (11).

Ismail et al. (28) showed how, in the hands of expert U-VATS surgeons, the mean number of lymph nodes retrieved during U-VATS lobectomy can be satisfactory (20.14 ± 10.73) and comparable to the mean reported in the literature for other techniques (13.42 ± 8.24 in Triportal-VATS, 9.44 in open surgery, 17 in RATS), as well as the incidence of nodal upstaging (13.3% in U-VATS vs. 6.7–12.8% reported in other approaches). In particular, the pN1-upstaging (7.4%) and pN2- upstaging (3%) were also in line with the literature (27).

### Nodal Upstaging and Surgical Outcomes

Till now, no study compared the nodal upstaging in U-VATS and open technique, considered the gold standard.

In our study, we tried to address this point, finding out no difference in nodal upstaging between the open and U-VATS groups after propensity score matching.

In particular, U-VATS surgery seems to be as effective as thoracotomy for detecting nodal upstaging [11 (23.9%) cases (9 were pN1-upstaging) after thoracotomy and 8 (17.4%) cases (5 pN1-upstaging) after U-VATS, *p*: 0.440]. N2-LNR seems to be improved in the U-VATS approach but this result must be confirmed in a larger population.

Boada et al. (29) argued that the type of approach chosen by surgeons is often influenced by the dimension and centrality of the lesions (5.5% in VATS group vs. 23.1% in open, *p*: 0.005): the larger and more central the tumor is, the more often the operation is conducted with an open technique. We found the same correlation as Boada et al. (29) in the whole population, with larger tumors (*p*: 0.030) or central tumors (*p*: 0.066) operated on by an open approach (Table 1). However, in our study, surgical technique or tumor dimension/centrality were not found to be risk factors for nodal upstaging after regression analysis neither in the whole population nor in the matched one.

About the other surgical outcomes, our study showed how the U-VATS approach seems to guarantee similar 3-year OS (78 vs. 74%, *p*: 0.204), DSS (97 vs. 89%, *p*: 0.371), and DFS (62 vs. 66%, *p*: 0.917) as open surgery but with a shorter chest tube duration (*p*:0.025) and lower post-operative (*p* << 0.001) and chronic (*p* << 0.001) pain. These survival results after U-VATS surgery are in line with those reached by previously published data on multi portal VATS compared to thoracotomy (25, 29). Another important aspect of our study conclusions is that U-VATS lobectomy seems to be not inferior compared to thoracotomy regarding short-term survival outcomes, and better for surgical results in stage I NSCLC, even including the learning curve period of the two first operators. This could endorse the theory about the supposed shorter learning curve of the U-VATS approach compared to other techniques for surgeons with a large experience in open surgery, thanks to U-VATS' direct visualization of the target as in open surgery and greater ergonomics, as stated before (16–18).

Our study has some important limitations. Many of the selection biases of the patients are related to the retrospective nature of the study. The number of patients involved is limited and belonged to a single institution. Furthermore, the mean oncological follow-up is shorter than 5 years and longer follow-up would be necessary to state long-term oncological outcomes. However, to the best of our knowledge, this represents the first study comparing U-VATS' effectiveness with traditional open surgery and showing its non-inferiority in terms of surgical outcomes, nodal upstaging rates, and short-term survivals.

With the limits indicated above, U-VATS surgery seems to be as safe and effective as thoracotomy in performing early-stage lung cancer lobectomy, guaranteeing the same nodal upstaging rates and short-term survivals but shorter chest tube duration, post-operative stay, and pain.

## Data Availability Statement

The raw data supporting the conclusions of this article will be made available by the authors, without undue reservation.

## Ethics Statement

The studies involving human participants were reviewed and approved by Ethics Committee of Università Cattolica del Sacro Cuore, Rome, Italy. The patients/participants provided their written informed consent to participate in this study.

## Author Contributions

DN and EM: study design, manuscript writing, and critical revision of the manuscript. MC, MV, MI, DT, CS, GC, and LP-C: data collection and manuscript writing. DN: statistical analysis. SM and EM: supervision. All authors contributed to the article and approved the submitted version.

## Conflict of Interest

The authors declare that the research was conducted in the absence of any commercial or financial relationships that could be construed as a potential conflict of interest.

## Publisher's Note

All claims expressed in this article are solely those of the authors and do not necessarily represent those of their affiliated organizations, or those of the publisher, the editors and the reviewers. Any product that may be evaluated in this article, or claim that may be made by its manufacturer, is not guaranteed or endorsed by the publisher.
